# Recent Developments in Differentiation Therapy of Acute Myeloid Leukemia

**DOI:** 10.3390/cancers17071141

**Published:** 2025-03-28

**Authors:** Ugo Testa, Germana Castelli, Elvira Pelosi

**Affiliations:** Department of Oncology, Istituto Superiore di Sanità, 00161 Rome, Italy; germana.castelli@iss.it (G.C.); elvira.pelosi@iss.it (E.P.)

**Keywords:** leukemia, acute myeloid leukemia, differentiation, targeted therapy, genetic abnormalities

## Abstract

Acute myeloid leukemia (AML) is characterized by the clonal expansion of myeloid progenitors blocked at various stages of their differentiation process, and drugs that bypass this differentiation block are therapeutically efficient, as shown by retinoic acid and arsenic trioxide in acute promyelocytic leukemia. However, the successful application of differentiation therapy in APL has not translated into clinical benefit for other non-APL subtypes of AML, in which intensive chemotherapy regimens represent the standard of care. However, the development of molecular studies has led to the identification of therapeutic targets (such as mutated proteins and deregulated pathways) and has led to the generation of a new category of specific pharmacologic agents. Some of these agents, such as inhibitors of mutant isocitrate dehydrogenase (IDH1 and IDH2), lysine-specific demethylase-1 (LSD1), and Menin, have shown the capacity to induce leukemic cell differentiation and with significant therapeutic efficacy.

## Simple Summary

Acute myeloid leukemia (AML) is a heterogenous disease caused by a combination of molecular events in hematopoietic stem cells that drive proliferation and block differentiation. The traditional approach to treating AMLs consists of inducing the large-scale elimination of leukemic blasts with chemotherapeutic agents. An alternative approach consists of inducing the differentiation of leukemic cells and has been successfully developed in the treatment of acute promyelocytic leukemia. For many years, the extension of differentiation therapy to other AML subtypes has remained challenging. However, the development of studies on the molecular characterization of AMLs has led to the identification of genetic abnormalities that can be specifically targeted by some drugs, such as inhibitors of isocitrate dehydrogenase (IDH), lysine demethylase 1 (LSD1), and Menin, capable of inducing AML differentiation. This review will discuss the clinical evolution of these inhibitors, outcome data from the latest clinical studies, the development of resistance mechanisms, and strategies to improve outcomes and future directions.

## 1. Introduction

A hallmark of acute myeloid leukemia (AML) is represented by a differentiation block of myeloid progenitors/precursors that accumulate in bone marrow and in the blood.

One AML subtype, acute promyelocytic leukemia (APL), displays a unique sensitivity to retinoic acid, promoting the differentiation of leukemic promyelocytes to mature neutrophils [1]. This discovery has provided the rationale for initial clinical studies based on the use of all-trans retinoic acid (ATRA) in APL, which has supported the capacity of this drug to promote the in vivo differentiation of leukemic cells [2,3,4]. Subsequent studies have clarified the molecular basis of the high sensitivity of APL to ATRA; in fact, APL is characterized by a 15;17 chromosome translocation with breakpoints within the retinoic acid receptor α (RARA) gene on chromosome 17 and the PML gene, which encodes a transcription factor on chromosome 15. A PML-RARA fusion protein is formed because of the translocation. The specific targeting of this protein represents the molecular basis of the marked sensitivity of APL to ATRA [5]. The PML-RARA protein is responsible for all of the unique features of APL cells, such as the sensitivity to ATRA, block of cell differentiation at the promyelocyte stage, and increased proliferation due to diminished apoptotic cell death [6]. This specific molecular vulnerability of APL has been exploited and allowed for the development of a curative dual differentiation therapy based on the association of ATRA and arsenic trioxide (ATO), leading to more than 90% of patients being cured from this leukemia without the use of cytotoxic chemotherapy [7].

Although differentiation therapy has completely revolutionized APL treatment, changing the outcome of this leukemic subtype from highly fatal to curable, significant challenges have been observed in attempting to develop differentiation-based treatments for other AML subtypes. However, some recent developments of targeted therapies have led to the identification of newer targets for differentiation therapy. Particularly, in this context, the targeting of some molecules in non-APL AMLs, such as isocitrate dehydrogenase 1 and 2 (IDH1 and IDH2), lysine-specific demethylase 1 (LSD1), and Menin, is associated with the induction of leukemic cell differentiation and significant therapeutic effects.

This review will analyze recent developments in the therapy of AML based on IDH, LSD1, and Menin inhibitors evaluated in clinical trials (Table 1).

## 2. IDH Inhibitors

### 2.1. Biology and Mechanisms of IDH Mutations

Isocitrate dehydrogenase (IDH) is an enzyme that catalyzes the oxidative decarboxylation of isocitrate, producing α-ketoglutarate and CO_2_. In humans, three isoforms of IDH exist: IDH3 catalyzes the third step of the citric cycle, while converting NAD to NADH in the mitochondria; the isoforms 1 and 2 (IDH1 and IDH2) catalyze the same reaction outside of the citric acid cycle and use NADP as cofactor.

Mutations of IDH are frequently observed in AML and their prevalence increases with age. While WT-IDH1 and IDH2 catalyze the decarboxylation of isocitrate to α-ketoglutarate, mutant IDH1 and IDH2 enzymes catalyze the conversion of α-KG to 2-hydroxyglutarate (2-HG), an oncometabolite responsible for the oncogenic activity of *IDH1* and *IDH2* mutations [8]. Cancer-associated IDH mutations occur at the level of distinct arginine residues in the enzyme active sites. IDH1/IDH2 mutations occur in about 20% of adult AML patients, with a higher frequency of *IDH2* (8–19%) than *IDH1* (6–16%) mutations. *IDH1* is most frequently mutated at the level of the Arg residue (R132), with R132H and R132C being the most recurrent *IDH1* mutations and R132L and R132S less commonly observed [8]. At the level of the *IDH2* gene, the most recurrent mutations occur at the level of Arg140 (R140Q) and more rarely at the level of R172 (R172K) [8].

Inhibitors of IDH are small molecules that bind within the IDH enzymatic active site, blocking aberrant 2-HG production and inducing myeloid differentiation.

A large study involving the molecular characterization of 1023 older AML patients reported a frequency of 9.7% for *IDH1^MUT^* and 18.9% for *IDH2^MUT^*, including 1% for double-mutant *IDH1/IDH2; IDH1^MUT^* significantly co-occurred with *DNMT3A^MUT^* (42.4%), while *IDH2^MUT^* was associated with *DNMT3A^MUT^* (35.8%), *NPM1^MUT^* (31.1%), and *SRFSF2^MUT^* (38.3%) [9]. *IDH1^MUT^* was less frequently associated with *TET2^MUT^* (6.3%) and *TP53^MUT^* (9.3%) [9]. A normal karyotype was more frequently observed in patients with *IDH1^MUT^* and *IDH2^MUT^*, while a complex karyotype was less frequent in patients with *IDH1^MUT^* and *IDH2^MUT^* AMLs [9]. Patients with *IDH2^MUT^* AMLs exhibited improved survival when treated with low-intensity treatments compared to those with *IDH^WT^* AMLs.

The co-mutation profile significantly differed in patients with *IDH1^R132C^* and *IDH1^R132H^* AMLs in that in the former ones, there was a significantly higher frequency of *SRSF2*, *ASXL1,* and *RUNX1* mutations and a lower frequency of *NPM1* mutations than in the latter ones [10].

The frequency of both *IDH1* and *IDH2* mutations in AML is strongly influenced by age. *IDH1 + IDH2* mutations increased from 3.4%, 11.3%, and 17.7% up to 21% in groups of pediatric (0–12 yrs), 13–39 yr old, 40–59 yr old, and >60 yr old patients, respectively [11]. *ASXL1* and *RUNX1* mutations were markedly increased in older AML patients [11].

Experimental studies have, in part, elucidated the mechanisms through which *IDH* mutations exert a leukemogenic effect. Studies in mouse models have shown that *IDH* mutations alone cannot induce the development of the full leukemic process in vivo but need to cooperate with additional genetic lesions to initiate leukemia [12,13]. More recently, transgenic *IDH2^MUT^* zebrafish models have confirmed these findings and have reported, in transgenic embryos co-expressing *IDH2* mutations and *FLT3^ITD^*, the development of a leukemic process recapitulating features of human *IDH^MUT^* AML [14]. A single-cell transcriptomic analysis showed increased myeloid skewing, differentiation blockade, and leukemia-associated gene signatures [14].

In vitro models of IDH mutations have shown a consistent pattern of DNA hypermethylation, similar to that observed in primary *IDH^MUT^* AML cells [15]. Conditional knock-in experiments have allowed for the exploration of the effects of DNA hypermethylation in hematopoietic progenitor cells (HPCs); using this approach, an *IDH1* mutation (*IDH1^R132H^*) was inserted into the endogenous murine *IDH1* locus and was expressed in all hematopoietic cells or specifically in cells of myeloid cell lineage [15]. These mutant mice showed increased numbers of HPCs and self-renewal capacity [12]. The expansion of HPCs and increased self-renewal were observed also in a model of transgenic mice expressing *IDH2^R140Q^* in the hematopoietic system; in addition, a block of cell differentiation was also observed [13].

In addition to hematopoietic cells, *IDH* mutations impair histone demethylation and inhibit cell differentiation in normal astrocytes [16].

Additional studies have shown that the *IDH1^R132H^* mutant promotes cytokine independence and blocks differentiation in hematopoietic cells and these effects can be recapitulated by 2-HG [17].

The key role of *IDH* mutations in blocking HPC differentiation and promoting leukemic growth was further and more strongly supported by the observation that a small molecule (AGI-6780) acting as a potent and specific inhibitor of mutant *IDH2* suppressed the growth of patient-derived leukemia cells and induced cell differentiation of *IDH2^MUT^* leukemic cells [18].

A recent study has shown that the expression of *IDH1^R132H^* and *IDH^R140Q^* mutants into human CD34^+^ cells via lentiviral transduction markedly impaired the colony forming capacity of erythroid, monocytic, and granulocytic lineage cells [19]. In line with this observation, CD34^+^ cells isolated from an *IDH2^MUT^* AML patient undergoing treatment with Enasidenib, an IDH2 inhibitor, showed a progressive improvement in their differentiation capacity, as supported by the in vitro generation of colonies of mature hematopoietic cells [19].

Finally, a recent study has additionally supported the role of *IDH* mutations in blocking hematopoietic differentiation. Landberg et al. generated CD34^+^ cells edited by CRISPR/Cas9 and AAV6-mediated homology-directed repair to express *IDH1^R132H^* or *IDH^R140Q^* mutations; the mutant CD34^+^ cells displayed a pronounced decrease in their colony and differentiation capacity. The block of cell differentiation of *IDH1^R132H^*-edited cells was rescued by Ivosidenib [20,21].

All of these observations support a key role of *IDH* mutations in blocking the cell differentiation of *IDH^MUT^* AMLs.

**Figure 1:**
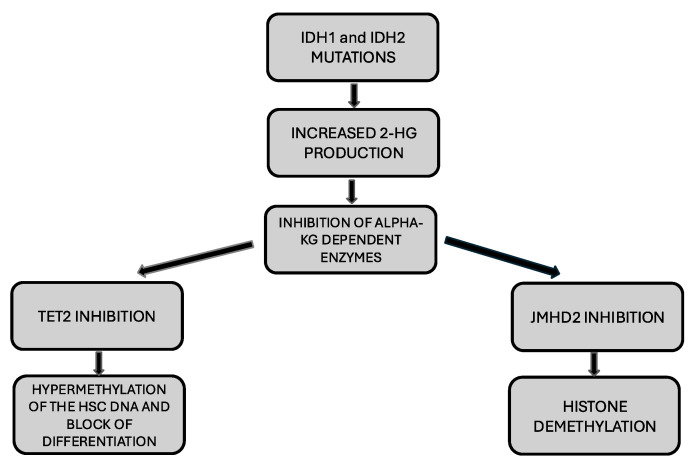
Various steps involved in the development of *IDH*-mutant AMLs, in relation to the pathogenetic contribution of *IDH1* and *IDH2* mutations.

It is important to underline that the effects of *IDH* mutations on cell differentiation are largely dependent on the methylation changes induced by these mutations. The effects induced by *IDH* mutations on DNA methylation are largely mediated by 2-HG: 2-HG competitively binds and inhibits α-KG-dependent enzymes such as ten-eleven translocation 2 (TET2), an enzyme converting 5-methylcytosine (5-mC) to 5-hydroxymethylcytosine (5hmC). 2-HG-mediated block of TET2 leads to the hypermethylation of the HSC genome [15] (Figure 1). *IDH1* and *IDH2* mutations in AML are associated with increased DNA methylation. The effects of these DNA methylation changes are not widespread but occur at the level of thousands of focal regions that are specifically hypermethylated compared to normal CD34^+^ cells and AMLs without *IDH* mutations [22]. The methylation profile observed at these focal regions is different compared to that observed at the level of loci commonly hypermethylated in AML [22]. The profile of hypermethylated loci is highly comparable in *IDH1*- and *IDH2*-mutant AMLs, but DNA hypermethylation is more pronounced in *IDH1^MUT^* AMLs than in *IDH2^MUT^* AMLs [22]. AMLs with biallelic inactivating *TET2* mutations had a less dramatic methylation phenotype, but many of the hypermethylated regions correspond to those observed in *IDH^MUT^* AMLs [22]. The 5 hydroxy methyl cytosine (5hmC) levels in hypermethylated DNA regions are significantly lower in *IDH^MUT^* and *TET2^MUT^* AMLs, thus providing evidence that both mutations lead to increased DNA methylation by blocking TET2-mediated demethylation (TET2 promotes DNA demethylation through the hydroxylation of 5mC) [22]. DNA hypermethylation in *IDH^MUT^* AML cells requires the activity of the DNMT3A metyltransferase. Importantly, *IDH^MUT^*-specific DNA hypermethylated regions are enriched at the level of enhancers involved in the interaction with genes involved in normal hematopoiesis and AML [22].

### 2.2. Small Molecule Inhibitors of IDH1: Ivosidenib

Ivosidenib (AG-120) is a first-in-class inhibitor of *IDH1^MUT^* that exhibits marked 2-HG reduction in *IDH1^MUT^* AMLs and the induction of cell differentiation [23]. Particularly, Ivosidenib inhibits R132 *IDH1* mutants with an IC_50_ in the range of 10–12 nM and, at higher concentrations, inhibits *IDH1-WT* but not *IDH2* [23].

### 2.3. Clinical Studies with Ivosidenib

Several clinical studies have explored the safety and efficacy of Ivosidenib alone or in association with other antileukemic drugs in IDH1-mutant AMLs (Table 2). A pivotal study by Di Nardo and coworkers reported a rate of complete remission (CR) or complete remission with partial hematological recovery (CRi) of 30.4%, with an overall response rate (ORR) of 41.6% and a median duration of response (mDR) of 8.2 months [24]. Bone marrow studies showed that Ivosidenib induced myeloid differentiation and hematopoietic recovery without an intervening period of bone marrow aplasia, a finding consistent with a mechanism of action involving the induction of myeloid cell differentiation [24]. Differentiation syndrome was observed in 11% of the treated patients and all of these patients showed different degrees of response to treatment [24].

Seventeen patients enrolled in this study responded to Ivosidenib treatment and subsequently underwent allogeneic HSCT [25]. A proportion of 12% of these patients had relapsed following prior transplant before starting Ivosidenib therapy, and 76% were refractory to initial therapy; the most common baseline co-mutations were *NPM1* (29%), *DNMT3A* (24%), and *SRFSF2* (18%) [24]. The prior responses to Ivosidenib before allo-HSCT were CR in 10/17, CRi in 4/7, and MLFS (morphologic leukemia-free state) in 3/17 [25]. The post-HSCT mOS was 7.7 months, with survival rates of 76.5% and 47.1% at 6 and 12 months, respectively; the mRFS was 7.3 months, with RFS rates of 58.8% and 47.1% at 6 and 12 months [23]. In all of the patients whose best OR was CR following Ivosidenib therapy and who underwent HSCT, the median OS was not reached [25].

In newly diagnosed *IDH1^MUT^* AML patients ineligible for standard chemotherapy induction, Ivosidenib treatment achieved a CR/CRi rate of 42.4%; 18% of the treated patients developed a DS [26].

The phase III trial AG120-C-009 evaluated newly diagnosed *IDH1^MUT^* AML patients ineligible for intensive chemotherapy receiving Ivosidenib and Azacitidine, a hypomettylating agent (IA), or placebo and Azacitidine (PA): the event-free survival was significantly longer in patients receiving IA than those receiving PA; the median OS was 24 months with IA and 7.9 months with PA [27]. The IA group had a favorable toxicity profile compared to the PA group [27]. DS was observed in 14% of the patients receiving IA and 8% in those treated with PA [27]. On May 2022, on the basis of the results of this trial, the FDA approved a supplemental application for Ivosidenib, extending the indication in patients with newly diagnosed *IDH1*-mutated AML patients in older adults or those with comorbidities to include the combination with azacitidine [28].

Several studies combining Ivosidenib- and Ventoclax-based regimens are under evaluation. In a phase IB trial, Ivosidenib and Venetoclax (IVO + VEN) with or without Azacitidine (AZA) were evaluated in 31 patients with IDH1-mutant AML. The CR + CRi rate was 90% in the patients treated with the triplet combination, compared with 83% in those treated with IVO + VEN alone [29]. A proportion of 63% of the patients attained an MRD-negative CR [29]. An updated analysis of this study, including 25 additional patients in phase II (all treated with the triplet drug combination) was recently reported, showing an ORR of 94% with a 93% CR + CRi; the 3-year OS was 70.5%; the patients who received HSCT had a 3-year OS of 94.7% compared to 52.8% in those who did not [30]. A retrospective study analyzed 283 newly diagnosed AML patients ineligible for intensive chemotherapy treated with Ivosidenib plus a hypomethylating agent (IVO + HMA) or VEN + HMA: the CRRs including CR + CRi were 63.3% and 49.5% for IVO + HMA and VEN + HMA, respectively; the 6-month EFS was 56% vs. 39.6% for IVO + HMA compared to VEN + HMA, respectively; and 11.5% of the patients with IVO + HMA compared to 5.0% of the patients treated with VEN + HMA were bridged to HSCT [31].

A phase I clinical study evaluated the safety and efficacy of Ivosidenib in association with induction chemotherapy in newly diagnosed *IDH1^MUT^* AML patients, resulting in a CR + CRi rate of 72% [32]. In this study, Ivosidenib was administered during induction, consolidation, and maintenance therapy; thus, it is difficult to understand the relative impact of Ivosidenib at each treatment stage. In this clinical setting, the frequency of DS was low [32]. A more careful examination of these patients at the level of the BM morphology and flow cytometric phenotype at day 14 and 21 showed three different patterns of response: (i) aplasia pattern of <10% cellular, <5% blasts; (ii) >10% cellular, <5% blasts, with morphologic and flow cytometric evidence of blast cell differentiation; (iii) and persistence of leukemic blasts [33]. A differentiation response was observed in 20% of the patients treated with Ivosidenib and chemotherapy [33].

Fathi et al. reported the results of a multicenter phase I trial involving post-HSCT treatment with Ivosidenib for *IDH1*-mutated AML [34]. In total, 16 patients were enrolled and 8 discontinued maintenance with Ivosidenib. The two-year PFS was 81% and the two-year OS was 88% [34].

The analysis of the primary response and relapse after Ivosidenib treatment allowed the researchers to define the mechanisms of resistance and relapse in R/R *IDH1^MUT^* AML patients. Clinical response to Ivosidenib was not predicted by the position of *IDH1^MUT^* within the clonal hierarchy: *IDH1^MUT^* was subclonal in 28% of cases and clonal in 72% of cases and there was no association between *IDH1^MUT^* subclonal or clonal status and the response to Ivosidenib [35]. Baseline RTK mutations (*NRAS*, *KRAS*, *FLT3-ITD*, and *FLT3-TKD*) are associated with primary treatment resistance [35]. Relapse is characterized by multiple mechanisms, including the emergence of RTK pathway mutations and *IDH* mutations conferring resistance to Ivosidenib [35].

The TARGET-seq^+^ method was used for single-cell genotyping to explore sequential bone marrow samples from eight patients (six relapsed and two in sustained remission), six treated with Ivosidenib/Venetoclax and two with Ivosidenib/Venetoclax/Azacitidine [32]. Relapse was associated with either genetic clonal evolution or impaired differentiation of pre-existing clones fully differentiated into mature myeloid cells prior to treatment within HSPC/precursor cell compartments known to demonstrate LSC potential [36]. In the two patients that remained in sustained remission, therapy eradicated all leukemic clones within three cycles of treatment [36].

In conclusion, clinical studies carried out using Ivosidenib have supported its target-specific antileukemic efficacy, associated with a good safety profile. However, several important questions remain to be answered in future studies: the optimal sequencing of Ivosidenib during the various stages of AML disease is unclear; the IVO + AZA regimen must be compared to the NEN + AZA regimen in prospective, randomized clinical trials; the triple therapy regimen (IVO + AZA + VEN) must be evaluated in randomized clinical trials with adequate controls; and the optimal combination therapy in R/R AML patients needs to be established.

### 2.4. Small Molecule Inhibitors of IDH1: Olutasidenib

Olutasidenib is an allosteric, highly selective, potent, orally administered *IDH1^MUT^* inhibitor that binds at the level of the hydrophobic pocket located near the IDH1 heterodimer interfaces and determines the stabilization of the mutant IDH1 molecule in an inactive conformation, thus blocking its neomorphic enzymatic activity. At the structural level, Olutasidenib is a quinoline derivative [37]. In contrast to Ivosidenib, Olutasidenib binds mutated *IDH1* in a 2:1 stechiometric ratio, thus offering a potential mechanism to overcome some mutations associated with Ivosidenib resistance [37]. Given these inhibitory properties, Olutasidenib inhibits 2-HG production and promotes granulo-monocytic differentiation in primary *IDH1^MUT^* AML cells and inhibits the growth of *IDH1^MUT^* AML xenograft models [38].

### 2.5. Clinical Studies with Olutasidenib

A phase I/II clinical study evaluated the safety and the efficacy of Olutasidenib in *IDH1^MUT^* AML and MDS patients. Phase I of the study evaluated the safety and efficacy of Olutasidenib as a single agent and in combination with azacitidine in patients with *IDH1^MUT^* AML and MDS; Olutasidenib monotherapy in R/R patients and Olutasidenib as monotherapy or in combination with Azacitidine was evaluated. Grade 3–4 hematologic adverse events were more common with combination therapy than with monotherapy; differentiation syndrome was observed in both the monotherapy and combination treatment [39]. The ORR was 41% in the patients receiving monotherapy and 46% in the patients treated with combination therapy; for treatment-naïve AML patients, the ORR was 25% in monotherapy and 77% in combination therapy [39]. For the R/R *IDH1^MUT^* AML patients, the median OS was 8.7 months for monotherapy and 12.1 months for combination therapy [39].

The phase II study involving monotherapy treatment enrolled 153 R/R *IDH1^MUT^* AML patients, with a median age of 71 years and a median of two prior therapy regimens; the CR + CRi was 35% and the ORR was 48%; the median duration of the CR + CRi was 25.9 months; the median OS was 11.6 months; and the response rates were similar in the patients who did and did not receive prior Venetoclax treatment [40]. OS occurred in 22% of the patients and 9% of the patients were grade ≥3, with one fatal case [40]. The final analysis at the 5-year follow-up showed a CR + CRi rate of 35% with a median duration of CR + CRi of 25.3 months; the ORR was 48%, with a median duration of 15.5 months; the mOS was 11.6 months and in the patients R/R to prior Venetoclax, the mOS was 16.2 months [41]. Transfusion independence from red blood cells and platelets was achieved in 39% and 41% of the patients, respectively [41]. The analysis restricted to elderly patients (≥75 years) showed that Olutasedinib was well tolerated and 31% of these patients achieved CR + CRi, with a median time to CR + CRi of 15 months and with a median duration of CR of 25.9 months [42].

Phase II of the combination of Olutasidenib and Azacitidine was based on a study involving four cohorts of patients: treatment-naïve patients with AML; R/R AML and MDS patients with no prior exposure to HMA or IDH1 inhibitors; patients R/R to HMA; and patients with prior exposure to IDH1 inhibitors [43]. In an initial report, 72 patients with AML/MDS were reported (20 R/R without prior HMA/HD1 inhibitor therapy; 21 R/R with prior HMA therapy; 20 R/R with prior HD1 therapy; and 11 treatment-naïve AML patients) [40]. In the treatment-naïve AML patients, the CR + CRi rates were 45% and in the R/R setting, the CR + CRi rates were 47% in those without prior HMA/HD1 inhibitor therapy, 38% with prior HMA/HD1 inhibitor therapy, and 30% with prior IDH1 inhibitor therapy [40]. The duration of CR + CRi was longest among the treatment-naïve patients [43].

More recently, a pooled analysis of R/R IDH1-mutant AML patients included in this trial was presented: 43% were refractory and 57% relapsed after a prior treatment; 83% of these patients had at least two prior treatment regimens, including HMA (40%), an IDH1 inhibitor (31%), and HSCT (10%); CR + CRi was observed in 31% of the patients, with a median duration of 15 months; CR was achieved in 27% of the patients with a median duration of 20 months; the ORR was 51%; the median OS was 13 months for all of the patients, being 24 months in the overall responders, 30.6 months in the patients with CR + CRi, and not reached in the patients with CR [44,45].

A multicenter, investigator-initiated phase I/II study will evaluate the safety and efficacy of a triplet regimen based on Olutasidenib in combination with Decitabine and Venetoclax [46]. Interestingly, Cortes et al. showed the efficacy and the safety of Olutasidenib administered to 18 patients with *IDH1*-mutant AML who relapsed or were refractory to a Venetoclax regimen; 31.3% of these patients displayed CR + CRi [47].

The outcomes in Olutasidenib-treated patients from the 2102-HEM-101 single-arm trial were compared to a real-world external control arm of Ivosidenib, suggestive of favorable effectiveness of Olutasidenib for patients with *IDH1*-mutant AML who are R/R to a Venetoclax-based regimen [48].

A recent study reported a comparative analysis of Olutasidenib and Ivosidenib. Both these drugs have been approved by the FDA for the treatment of IDH1-mutant AML. In the absence of head-to-head studies, this analysis compared the outcomes observed in studies carried out in R/R AML patients: the rates of CR + CRi observed with Olutasidenib and Ivosidenib were similar; however, the duration of the response observed in the patients treated with Olutasidenib was longer than that observed in patients treated with Ivosidenib (25.9 months vs. 8.2 months, respectively). Another notable difference is that Olutasidenib is structurally smaller with a lower molecular weight than Ivosidenib; thus, it occupies less space in the binding pocket of IDH1 dimers, making it resistant to displacement by a second IDH1 mutation [49]. In biochemical studies, Olutasidenib was found to inhibit mutant *IDH1* but not wild-type IDH1 [49].

In conclusion, clinical studies have supported a favorable safety profile and significant antileukemic activity of Olutasidenib. As outlined above, only indirect comparisons with Ivosidenib have suggested that Olutasidenib could induce a longer duration of remission than Ivosidenib. Furthermore, future studies are required to evaluate Olutasidenib as a salvage therapy in patients failing after Ivosidenib treatment. Finally, combination treatments in regimens including Venetoclax have to be explored both in newly diagnosed and R/R AML patients.

### 2.6. Small Molecule Inhibitor of IDH2: Enasidenib

Enasidenib (AG-221) is a specific *IDH2*-mutant inhibitor developed by Agios Pharmaceuticals/Celgene, reducing 2HG levels and promoting cell differentiation [50]. Particularly, Enasidenib binds to an allosteric site within the enzyme dimer interface, stabilizing an open conformation of the *IDH2*-mutant enzyme and inhibiting the conversion of α-KG to 2HG [50]. Enasidenib potently inhibited 2HG production by *IDH2^R140Q^* homodimer and *IDH2^R140Q/WT^* heterodimer and displayed favorable pharmaceutical properties such as solubulity, slow clearance and oral bioavailability [50].

### 2.7. Clinical Studies with Enasidenib

Several clinical trials have explored the safety and the efficacy of Enasidenib alone or in association with other antileukemic drugs in *IDH2*-mutant AML patients (Table 3).

A pivotal study by Stein and coworkers in relapsed or refractory *IDH2* mutant AML patients reported an overall response rate of 40.3%, with a median OS of 9.3 months (19.7 months in patients who attained a CR); responses were associated with the cellular differentiation of leukemic blasts and cytotoxicity was not the main driver of the antileukemic activity of Enasidenib [51]. Enasidenib was also explored in older AML patients who were unfit for intensive chemotherapy, showing an ORR of 30.8%, with 18% achieving CR and with an mOS of 11.3 months [52]. The analysis of R/R AML patients treated with Enasidenib showed that Enasidenib induced a marked decrease in the 2-HG levels, preceding the clinical response; 2-HG suppression by Enasidenib did not predict response since most non-responding patients also exhibited 2-HG suppression; complete remission and the normalization of stem and progenitor compartments were associated with the emergence of functional neutrophils bearing *IDH2* mutations; in a subset of responding patients, the *IDH2^MUT^* burden decreased and remained undetectable during the response; co-mutations in NRAS and MAPK pathway effectors were enriched in the patients resistant to treatment [53].

A randomized phase 3 clinical trial explored Enasidenib vs. conventional care in a population of 319 older *IDH^MUT^ R/R* AML patients who received two to three prior AML-directed therapies [48]. Enasidenib improved the EFS and hematological parameters but failed to improve the OS compared to standard treatment; the OS could be confounded by early dropout and the use of subsequent AML therapies. [54].

In a phase Ib/II clinical trial, Enasidenib was evaluated in 101 newly diagnosed *IDH2*-mutated AML patients not eligible for standard induction chemotherapy, the treatment based on Enasidenib plus Azacitidine (two-thirds of patients), or Azacitidine alone (one-third of patients) [55]. Significant differences were observed between the two groups of patients concerning the ORR (48% vs. 14% in Ena + AZA vs. AZA alone, respectively) and CRR + CRRi (43% vs. 8% in Ena + AZA vs. AZA alone, respectively), but not in the median OS [55]. About one-third of the patients in the group receiving Azacitidine alone received Enasidenib at progression as a post-protocol therapy and this may have confounded the survival analysis [55].

Explorative correlative studies performed in patients treated with Enasidenib and Azacitidine showed that responding patients displayed a decrease in blast cell population markers and an increase in differentiated myeloid markers, such as CD11b, and these changes were paralleled by a decrease in the VAF of *IDH2* mutations or *NPM1* mutations up to low/very low levels; no patients with *NRAS*, *KRAS*, or *PTPN11* mutations achieved CR [56].

A recent study explored Enasidenib combined with Venetoclax in a population of patients with R/R AML or other myeloid malignancies, showing that this drug combination induced an ORR higher than that observed with Enasidenib alone: the ORR was significantly higher in the *IDH2^R172^-* than in the *IDH2^R140^*-mutant patients (83% vs. 55%, respectively) [57]. In the whole treated population, the ORR was 70% and the CRR was 57%; the median duration of response was 16.6 months, and three patients proceeded to HSCT following CR [57].

The combination of Enasidenib with intensive induction and consolidation chemotherapy was explored in 93 fit *IDH2^MUT^* AML patients, showing that it was well tolerated and was associated with a CR + CRi rate of 77%, with 23% of the patients achieving *IDH2* mutation clearance [32].

A recent pilot clinical trial (NCT 03728335) assessed the use of Enasidenib as a post-HSCT maintenance therapy for 15 patients with *IDH2*-mutated AML: the patients received 24 cycles of Enasidenib therapy with leukemia-free survival rates of 100%, chromic GVHD-free survival rates of 93%, and relapse-free survival rates of 87% [58]. At the safety level, the treatment was well tolerated, with adverse events easily managed [58]. The results were considered highly promising and the enrollment of 20 additional patients was scheduled [58].

Concurrent RAS-signaling mutations are a great challenge in the treatment of *IDH*-mutant AML patients in that they infer resistance to IDH inhibitors. Thus, as supported by preclinical studies, a phase Ib study was proposed based on the administration of Enasidenib and MEK inhibitor Cobimetinib in R/R AML patients who have co-occurring *IDH2* and *RAS* signaling mutations [59].

Interestingly, a recent study reported an ORR of 43% in high-risk *IDH2*-mutant MDS treated with Enasidenib monotherapy, with an OS of 14.9 and 25.5 months in R/R and first-line treated patients, respectively [60]. Durable responses were observed in >50% of the low-risk MDS patients [60].

In conclusion, Enasidenib showed a good safety profile and displayed a significant antileukemic efficacy. Although Enasidenib was approved for the treatment of R/R AML patients, a randomized phase III clinical study failed to show an improvement in the OS compared to that with the standard chemotherapy treatment. Similarly, Anasidenib in association with Azacitidine failed to improve the OS of newly diagnosed AML patients compared to Azacitidine alone. Ongoing clinical studies have shown promising preliminary results when Enasidenib was associated with standard chemotherapy or with Venetoclax; however, randomization studies are required to assess the real impact of Enasidenib in these clinical settings.

### 2.8. Differentiation Syndrome in Patients Treated with IDH Inhibitors

Treatment with IDH inhibitors was associated with the development of differentiation syndrome (DS), a potentially lethal adverse reaction triggered by agents that induce myeloid differentiation.

DS was initially described in acute promyelocytic leukemia (APL) treated with agents inducing myeloid differentiation, such as all-trans retinoic acid (ATRA) and arsenic trioxide (ATO) [61]. DS is a relatively common and potentially severe complication observed in APL patients treated with ATRA and/or ATO; this syndrome is characterized by the association of a number of symptoms, including unexplained fever, weight gain, dyspnea with pulmonary infiltrates, pleuropericardial effusion, hypertension, and renal failure [61,62]. This syndrome may occur in a mild and in a severe form. The mechanisms responsible for the development of DS remain not fully understood but seem to be related to the induction of the differentiation of leukemic blasts, resulting in the massive release of cytokines (“cytokine storm”) and chemokines, and a subsequent systemic inflammatory response and increased expression of adhesion molecules on the surface of differentiated leukemic cells, mediating their adhesion to vascular endothelium and then their migration into tissues [63].

The prevalence, risk factors, and clinical outcomes of *IDH-*mutant AML patients treated with IDH inhibitors were not prospectively evaluated. A retrospective analysis performed by an independent differentiation review committee, composed of investigators who have participated in the development of Enasidenib, showed that DS was observed in 11.7% of R/R AML patients treated with Enasidenib and that the most frequent symptoms were dyspnea, fever, pulmonary infiltrates, and hypoxia; in 395 of cases, DS was associated with concomitant leukocytosis [64].

The Food and Drug Administration performed a systematic analysis of DS observed in 393 AML patients treated with Ivosidenib or Enasidenib [60]. According to this analysis, DS was identified in 19% of the patients; the predominant symptoms observed in these patients are pulmonary (dyspnea, pulmonary infiltrates, and effusions) [65]. In these patients, the onset of symptoms occurred at a median of 19–20 days after treatment [61]. The predictors of DS were represented by a higher leukemic burden in the bone marrow (≥48% of blasts) and in the blood (≥15–25% blasts); furthermore, concurrent mutations in *TET2* and *SRFSF2* were associated with a higher risk of developing DS, and the CR + CRi rates were lower in the patients with versus those without DS (Ivosidenib 18% vs. 36%; Enasidenib 18% vs. 25%) [65].

Montesinos and coworkers performed a pooled analysis from four clinical trials involving Enasidenib in AML patients, either as a monotherapy or in combination with Azacitidine or with chemotherapy [66]. The highest incidence of DS was observed in the patients who received Enasidenib plus Azacitidine (17.6%) and the lowest incidence was seen in the patients who received Enasidenib plus chemotherapy (2.2%) [66]. The most common symptoms were dyspnea/hypoxia (80.6%) and pulmonary infiltrates (73.1%) [66]. The baseline risk factors for developing DS were represented by higher levels of bone marrow blasts and lactate dehydrogenase [66].

## 3. Inhibitors of Lysine-Specific Demethylase 1 (LSD1 or KMD1A)

### 3.1. Role of LSD1 Inhibitors in HSC Differentiation and in Myeloid Leukemia

LSD1 is a member of the flavin adenine dinucleotide-dependent (FAD-dependent) amine oxidase family of demethylase, participating in the formation of various chromatin multiprotein complexes involved in gene regulation, such as rest co-repressor (CoREST) and nucleosome and remodeling and deacetylase (NuRD) [67,68]. LSD1 acts by demethylating lysine 4 and 9 on histone 3 and also on non-histone substrates, such as p53 and, through these effects, it represses gene expression [67]. LSD1 is essential for the function of HSCs and its knockout results in a reduction in granulopoiesis, thrombocytopoiesis, and erythropoiesis, associated with an expansion of granulo-monocytic, megakaryocytic, and erythroid progenitors, thus supporting a physiological role for LSD1 in myeloid differentiation and maturation [69]. LSD1 is overexpressed in many cancer types, including AML [70].

### 3.2. Small Molecule Inhibitors of LSD1

A number of compounds with amine oxidase inhibitory activity (trancylcypromine, paragyline, and phenelzine) and SP-2509 and GSK-LSD1 have been utilized in initial studies aiming to explore their anticancer activity. New LDS1 inhibitors suitable for clinical studies, such as ORY-1001 (Iadademstat), SP-2577 (Seclidemstat), IMG-7289 (Bodemstat), and CC-90011 (Pulodenstat), have been evaluated in clinical studies [67,68]. These compounds act as irreversible or reversible LSD1 inhibitors with various degrees of specificity and display in vitro efficacy in AML with the inhibition of cell proliferation and induction of cell differentiation [67,68].

**Figure 2:**
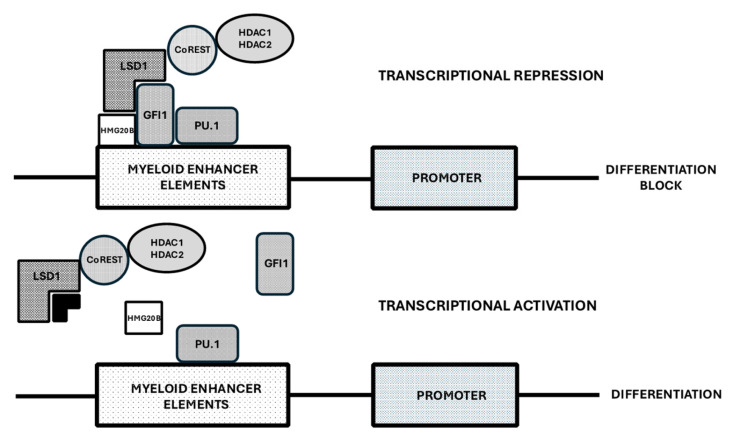
Molecular mechanisms underlying induction of cell differentiation by LSD1 inhibitors. LSD1 inhibition drives expression of genes involved in myeloid cell differentiation through disruption of transcriptional repression mediated by GFI1. *Top Panel*: when LSD1 interacts with the transcriptional repressor GFI1 and with HMG20B, it allows the recruitment of repressors to chromatin and catalyzes H3K4 demethylation and histone deacetylation though HDAC activity, resulting in transcriptional repression. *Bottom Panel*: LSD1 inhibitors block the interaction between LSD1 and GFi1, thus destabilizing the whole LSD1 repressor complex and leading to activation of myeloid enhancer elements with consequent transcriptional activation of master transcription factors of myeloid differentiation, such as PU.1.

Experimental studies in suitable models have supported the clinical use of LSD1 inhibitors as antileukemic drugs. A pivotal study by Lynch and coworkers provided evidence that pharmacological inhibitors of LSD1 promote the differentiation of myeloid leukemic cells through a mechanism independent of the inhibition of LSD1 enzymatic activity (histone demethylation) but dependent of the interaction between LSD1 and transcription factor GFI1, essential to maintain the differentiation block in AML [71]. Particularly, LSD1 inhibitors disrupt the interaction of LSD1 and RCOR1 with the transcription repressor GFI1, which is bound to a set of enhancers located close to transcription factor genes involved in the regulation of myeloid cell differentiation; the inactivation of GFI1 leads to increased enhancer histone acetylation and consequent gene activation [72]. LSD1 inhibitors interfere with the GFI-mediated repression of PU.1 and C/EBPα target genes and induce the differentiation of AML cells [73,74] (Figure 2). The stabilization of the binding of LSD1 on chromatin at GFI1 binding sites requires the interaction of the HMG-box protein HMG 20B with LSD1 [75] (Figure 2).

Cai et al. showed that the cell of origin of leukemic cell transformation seems to be a determinant of their sensitivity to LSD1 inhibitors [76]. Particularly, they showed that leukemias initiating in HSCs highly express the transcription factor EVI1, have attenuated p53 transcriptional output, are less sensitive to chemotherapy, and are resistant to LSD1 inhibitors [76]. P53 loss of function in progenitor-derived leukemias expressing low EVI1 levels induces resistance to LSD1 inhibition [76]. Interestingly, EVI1^high^ leukemias are sensitized to LSD1 by pretreatment with Venetoclax [76].

The LSD1/CoREST/HDCA2 complex is recruited by the SNAG domain of GFI1 [72]. In addition to GFI1, GFI1B is another repressor containing a SNAG domain and mediating the recruitment of LSD1/CoREST/HDCA2 complexes [77]. These molecular complexes involving LSD1 act as epigenetic regulators of hematopoietic differentiation [77]. GFI1 plays an important role in the control of granulo-monocytic differentiation, while GFI1B plays a role in erythroid and megakaryocytic differentiation [78].

The N-terminal intrinsically disordered region (IDR) is required for the regulation of LSD1–transcription factor interactions, controlling enhancer activation that is necessary for AML cell differentiation [79].

### 3.3. Clinical Trials with LSD1 Inhibitors

ORY-1001 is a potent and selective LSD1 inhibitor that induces H3K4me2 accumulation on LSD1 target genes, differentiation of leukemic cells, and inhibition of leukemic stem cell activity in AML models [80]. ORY-1001 synergizes with other antileukemic drugs currently used in the treatment of AML and, as seen in a patient-derived xenograft model of T-ALL, significantly reduces leukemia development and improves survival [80]. These observations have supported the clinical evaluation of ORY-1001 (Iadademstat) in AML patients (Table 4).

A phase I clinical study evaluated Iadademstat in relapsed or refractory AML patients: the dose-escalation section of the study carried out in 27 patients showed that the recommended dose of Iadademstat for an extension cohort was 140 μg/m^2^ administered as a single agent from day 1 to 5 of 28-day cycles; in the extension cohort, 14 additional patients were treated, including 5 patients with MLL/KMT2A-rearranged AMLs [81]. A reduction in the blood and bone marrow percentages of blasts was observed, with one patient achieving CR; the inhibition of blast cell differentiation was frequently observed, particularly in patients with MLL translocations: cell differentiation was observed in 80% of these patients and was particularly pronounced in two patients, with one of these patients developing a rapid and acute differentiation syndrome [81]. Preclinical studies have shown a synergism between Azacitidine and Iadademstat, thus supporting the clinical evaluation of this drug combination. Thus, the phase II ALICE clinical study evaluated 36 newly diagnosed AML patients with intermediate or advanced-risk disease, with a median follow-up of 22 months; dose–response studies showed that the optimal dose of Iadademstat in combination with Azacitidine was 90 μg/m^2^ [82]. In 27 patients, the ORR was 81% with 14/27 CR + CRi (11/27 MRD−) [82]. Three patients displayed DS and one patient had a fatal grade 5 intracranial hemorrhage (Table 4).

Iadademstat is also being evaluated in combination with Venetoclax and Azacitidine in newly diagnosed AML patients in an investigator-initiated phase I clinical trial at Oregon Health & Science University Knight Cancer Institute (NCT 06357182) and in a company-sponsored phase Ib clinical trial in combination with Gilteritinib in patients with R/R AML harboring *FLT3* mutations (NC 05546580).

Particularly, concerning the association of Iadademstat with Gilteritinib, the FRIDA study aims to establish the safety, tolerability, and recommended phase II dose in R/R *FLT3*-mutant AML patients [83]. This trial was supported by preclinical studies showing a strong synergy of Iadademstat with FLT3 inhibitors and particularly with Gilteritinib in *FLT3*-mutant AML cells [83]. The FRIDA study was based on a classical design for phase I/II studies, with an escalation phase (from 75 to 150 μg/orally of Iadademstat) and an expansion phase at the selected safe and pharmacologically active dose [83]. The preliminary results for the first 13 patients of the FRIDA trial were presented at the EHA 2024 Meeting: the combination of Iadademstat and Gilteritinib appeared to be safe and well tolerated, with no dose-limiting toxicities at the initial dose (75 μg) and DL1 (100 μg) of Iadademstat; encouraging antileukemic activity was shown, with 5 out of 13 patients (38%) achieving CR + CRi and 9 out of 13 patients (69%) achieving bone marrow blast clearance in the first cycle of treatment [84].

Two other LSD1 inhibitors, SP-2577 (Seclidemstat) and CC-90011 (Purodemstat), are under evaluation in patients with hematological malignancies, but their evaluation in AML patients is, at the moment, very limited.

In conclusion, the clinical studies carried out using Iadademstat in R/R AML patients have shown that this inhibitor has limited efficacy when used in monotherapy. However, the clinical studies performed in *FLT3*-mutated R/R AML patients using Iadademstat in association with Gilteritinib have preliminarily shown promising results that need to be extended and confirmed in randomized clinical trials with an appropriate control group. In newly diagnosed Aml patients, promising results were observed when Iadademstat was used in association with Azacitidine that need to be confirmed and validated in terms of their clinical impact through randomized clinical studies.

## 4. Menin Inhibitors

### 4.1. Role of Menin in the Control of Normal and Leukemic Hematopoiesis

The Menin protein is encoded by the *MEN 1* gene, whose germline mutations are the causation of sporadic or autosomal dominant hereditary cancer syndromes affecting the endocrine system; Menin plays a key role as an epigenetic regulator of gene expression for its role as a scaffold protein able to interact with various partners; thus, Menin acts as an adaptor protein between H3K4 (histone 3 protein with the lysine amino acid at position 4) methyltransferase KMT2A (also known as MLL), and LEDGF (lens epithelium derived growth factor). Menin interacts with both the wild-type and rearranged KMT2A, regardless of the fusion partner. Menin was found to be crucial for KMT2A activity and the maintenance of HOXA expression but not essential for normal hematopoiesis. Normal regulation of KMT2A activity is required for the maintenance of expression of the HOX family cluster genes in tissues.

Upregulated HOXA/MEIS1 expression is observed in AMLs characterized by the presence of *NPM1* mutations or by rearrangements of the *MLL* gene [78,79,80]. In *NPM1*-mutant AMLs (20–30% of total AMLs), the nucleolar protein is mutated and is usually delocalized in the cytoplasm; however, a part of the mutated NPM1 protein resides in the nucleus, where it interacts and is co-localized with KMT2A at the level of the *HOXA* locus [85,86,87]. The mutant NPM1c protein amplifies the function of KMT2A [79] and also inhibits the activity of histone deacetylases [87] to maintain the active transcription of *HOXA/B* cluster genes and *MEIS1*. Mutant *NPM1* upregulates the expression of *HOXA/B* gene clusters also through another mechanism dependent upon the interaction between mutant NPM1c protein and FOXM1 protein with its consequent inactivation and delocalization in the cytoplasm: the transcriptional inactivation of FOXM1 by cytoplasmic sequestration induces the de-repression of *HOX A/B* cluster genes [88].

*KMT2A*-rearranged AMLs represent 5–10% of adult AMLs, but are much more frequent in younger patients; in these leukemias, the rearrangement of the *KMT2A* gene upregulates HOX-MEIS1 expression [89]. The KMT2A protein directly binds to and uses its SET domain H3 (Lys4) methyltransferase to regulate *HOX* gene promoters. A third AML subset associated with increased HOX A/B expression is represented by the partial tandem duplication (PTD) of the *KMT2A* gene, occurring in 3–11% of adult de novo AML and associated with adverse outcomes. At the level of gene expression, *KMT2A-PTD* is characterized by a peculiar profile, showing the increased expression of several *HOX* genes, including *HOX-B5*, *HOX-B7*, *HOX-B8*, and *HOX-B9*. A comparative analysis of the profile of *HOX-A/B* genes deregulated in these AMLs overexpressing *HOX* genes showed a different profile in *KMT2A-PTD-* and *KMT2A*-rearranged AMLs and a similar profile to that of *KMT2A-PTD-* and *NPM1*-mutant AMLs [90]. The analysis of the whole gene expression profile allowed for the definition of three gene signatures along the HOX-targeted gene axis (HOX primitive, HOX transient, and HOX committed profiles); the *KMT2A*-rearranged AMLs were mostly classified as HOX-committed, while the *KMT2A-PTD* as HOX-primitive/transient and *NPM1*-mutant AMLs were distributed along the three HOX profiles [90].

A recent study provided an analysis of pediatric AMLs subdivided according to the *HOX* family gene expression. No expression or very low expression of the *HOXA* and *HOXB* genes was observed in normal bone marrow cells, while 25% of pediatric AML had significant upregulation of *HOX A/B* gene expression. The cluster of AMLs with the highest *HOX A* expression contained 88% of the *KMT2A*-rearranged AMLs and these patients displayed a significantly worse outcome compared to those with low/moderate *HOX* expression; patients without *KMT2A* rearrangements that clustered within the group at the highest *HOX A* expression had a dismal outcome [91]. Patients with high *HOXB* expression mainly have *FLT3-ITD* or *NUP98-NSD1* gene alterations; NUP-98-rearranged AML also expresses high *HOXA* levels [91]. AMLs with *HOX-A* expression display a clear co-expression of *MEIS-1* [91]. *NUP98* gene rearrangements are associated with increased *HOX* gene expression, related to a direct activation effect exerted by NUP98 fusion proteins [88,92].

In addition to *NUP98*, *NUP214* is another nucleoporin involved in translocations with two chromatin remodeling proteins, SET and DEK, resulting in fusion proteins that influence *HOX* gene expression [88,92].

### 4.2. Menin Inhibitors

**Figure 3:**
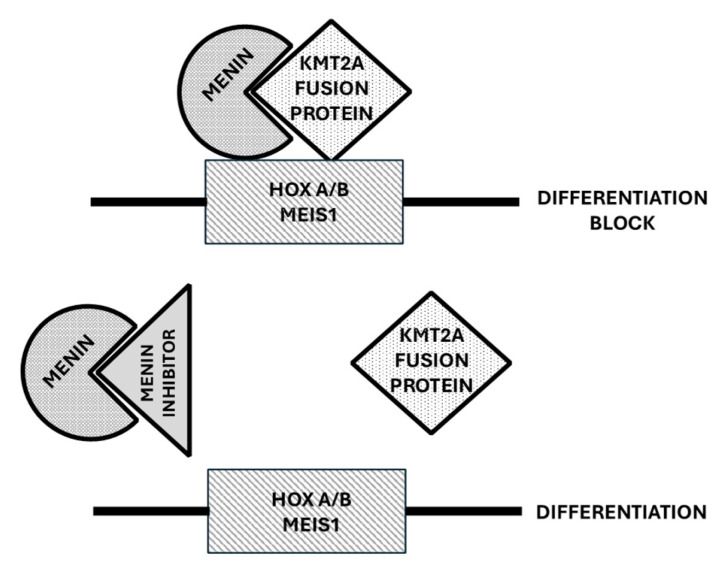
Role of Menin in the regulation of HOX A/B gene expression and cell differentiation in KMT2A-rearranged AMLs. *Top Panel:* KMT2A fusion protein binding to its cofactor Menin acts as a stimulator of HOX A7B gene expression, with consequent effects resulting in a block of cell differentiation. *Bottom Panel:* Menin inhibitors bind to Menin and impair its interaction with HOX A/B genes, inhibit HOX A/B gene expression, with consequent induction of cell differentiation.

By applying high-throughput screening, Grembecka and coworkers identified lead compounds targeting and inhibiting the Menin–MLL interaction; some of these compounds were then optimized by medicinal chemistry to develop inhibitors with nanomolar affinities [93,94]. Preclinical studies have shown that these compounds block proliferation, inducing apoptosis and differentiation in leukemic cells bearing MLL translocations; furthermore, these inhibitors reverse MLL fusion protein-mediated leukemic transformation though the downregulation of the expression of target genes involved in MLL fusion protein oncogenic activity [94] (Figure 3). Preclinical studies have supported the efficacy of Menin inhibitors in both *MLL*-rearranged and *NPM1*-mutated models of leukemia. Thus, Krivstov et al. showed that the highly selective Menin inhibitor VTP50469 displaced Menin from protein complexes and inhibited chromatin occupancy by *MLL* of selected genes in leukemic cells bearing *MLL* rearrangements, resulting in the induction of cell differentiation and apoptosis [94]. Patient-derived xenograft models derived from *MLL*-rearranged AML cells showed dramatic reductions in leukemia when treated with VTP50469 [95]. Klossowski et al. confirmed that the Menin-specific inhibitor MI-3454 inhibited cell proliferation and induced differentiation in primary patient samples with *MLL* translocations or *NPM1* mutations and induced the remission or regression of leukemia in mouse models of *MLL*-rearranged or *NPM1*-mutated leukemia [96]. Menin inhibitors silence both a canonical *HOX-* and *MEIS-1*-dependent oncogenic gene expression program and a noncanonical transcriptional program involving tumor suppressor genes and both these events are required to achieve a good therapeutic response [97].

In vitro studies in *NPM1*-mutant or *MLL*-rearranged leukemic cell lines or primary AML cells using the Menin inhibitor DS-1594b showed that the primary effect was the induction of cell differentiation and not of apoptosis; when the Menin inhibitor was associated with the BCL-2 inhibitor Venetoclax, the apoptotic effect was predominant [98].

### 4.3. Clinical Studies with Menin Inhibitors

#### 4.3.1. Revumenib

Revumenib is a potent oral small molecule inhibitor of the Menin–KMT2A interaction. This compound was evaluated in monotherapy in R/R AML patients with *KMT2A* rearrangements or *NPM1* mutations. The AUGMENT-101 trial (NCT 04065399) is a phase I clinical study evaluating the safety and efficacy of Revumenib in patients with R/R heavily pretreated *NPM1*-mutant or *KMT2A*-rearranged AMLs [88]. Since Revumenib is a substrate of CYP3A4, the study was subdivided into two cohorts: cohort A, with patients receiving a CYP3A4 inhibitor, and cohort B, with patients not receiving a CYP3A4 inhibitor [85]. The first evaluation of this study was based on 60 patients (46 with *KMT2A*-rearranged AMLs and 14 with *NPM1*-mutant AMLs); the ORR was 59% in the *KMT2A*-rearranged AMLs and 36% in the *NPM1*-mutant AMLs; the rate of CR + CRi was 30%; with a median follow-up of 11.9 months, in the patients who achieved morphologic CR + CRi, the median duration of response was 9.1 months; the mOS was 14.3 months; 56% of the responder patients achieved MRD negativity and 38% underwent allogeneic hematopoietic stem cell transplantation [99] (Table 5). Interestingly, in many patients with *KMT2A* rearrangements achieving morphological remission after one cycle of treatment, there was continued evidence of *KMT2A* fusions, a phenomenon seemingly related to the induction of leukemic blast differentiation elicited by Revumenib. Differentiation syndrome was observed in 16% of the patients, with all cases classified as grade 2 [99]. An additional eight AML patients with *NPM1-WT* and *KMT2A-WT* did not respond to Revumenib [99]. An analysis of the pharmacodynamic effects of Revumenib assessed through the transcriptional analysis of the RNA expression of bone marrow cells showed that Menin inhibition resulted in the downregulation of HOXA9 and MEIS1, associated with an increase in the expression of genes related to differentiation, such as CD11b and CD14 [99]. Single-cell studies carried out on four patients treated with Revumenib showed a differentiation continuum that started with immature AML blasts (CD34^+^/c-kit^+^), progressed through intermediate blast cells (CD68^+^, CD11b^−^, and CD14^−^), and ended with more differentiated monocytic cells (CD68^+^, CD11b^+^, and CD14^+^); intermediate AML blasts and monocytic cells were enriched in post-treatment samples [100].

A second study reported the results of phase II, carried out in 94 patients with R/R *KMT2A*-rearranged acute leukemia (78 with AML and 14 with ALL); 13% of the patients discontinued treatment for adverse events; DS was observed in 26% of the patients (14.9% had grade 3 and 1% grade 4) [101]. The CR + CRi rate was 22.8%, with an ORR of 63.2%; the median duration of response was 6.4 months; the mOS was 8.0 months; and among the responding patients, 38.9% received allo-HSCT [101]. Transcriptional changes in the bone marrow cells showed a decrease in MEIS1 and HOXA expression and an increase in the differentiation-related genes CD11b and CD14 [101]. An updated evaluation of the *KMT2A*-rearranged AML patients enrolled in the AUGMENT-001 trial extended to a total of 116 patients showed that 23% of the patients achieved CR + CRi, with an ORR of 64% and with a rate of 5.8% for MRD negativity among the patients achieving CR + CRi; 34% of the patients who achieved ORR proceeded to HSCT [102].

Recently, Syndex Pharmaceuticals announced the results of phase II of the AUGMENT-001 study evaluating the efficacy of Revumenib in 64 R/R heavily pretreated (75% relapsing after Ventoclax therapy) *NPM1*-mutated AML patients: 23% of the patients achieved CR + CRi; the ORR was 47%; the MRD negativity was 64% among the patients who achieved CR + CRi; and 17% of the responding patients underwent HSCT [103].

The ALLG AMLM26 INTERCEPT multiarm study was designed to obtain proof-of-concept for novel therapies targeting MRD or early relapse in AML [104]. In the context of this study, a preliminary analysis on nine *NPM1-*mutant AML patients with MRD-positivity showed an *NPM1* mutant ≥ 1 log_10_ MRD reduction in 62.5% of the patients, with 37.5% of the patients achieving MRD negativity [104].

A phase I/II study evaluated the combination of Revumenib with decitabine/cedazuridine and Venetoclax in R/R AML patients (*NPM1*-mutant, *KMT2A*-rearranged, and *NUP98-*rearranged), reporting an ORR of 88%, with a CR + CRi of 58%, and with a rate of MRD negativity according to flow cytometry of 93% among the patients with CR + CRi [105].

In pediatric patients, KMT2A rearrangements are frequently associated with RAS pathway mutations (51% of cases) and with a worse prognosis [106]. A recent preclinical study showed that the combination of a Menin inhibitor (VTP-50469, an analog of Revumenib) and Selutenib (a MEK 1–2 inhibitor) exerted a synergistic antitumor effect in vitro and in PDX models of *KMT2A*-rearranged AML cells bearing RAS pathway mutations [106].

About 40% of patients treated with Revumenib develop *MEN1* mutations and some acquire resistance to Menin inhibitors without *MEN1* mutations. Particularly, Perner et al. identified Menin gene mutations that were not present at diagnosis and developed with Revumenib treatment; the clonal expansion of these mutations was observed in about 39% of evaluable patients who had undergone at least two cycles of treatment [107]. These mutations were found at residues M327, G331, or T349 which do not impact the interaction between KMT2A and Menin or its oncogenic properties but decrease the binding affinity of Revumenib to KMT2A and mediate therapeutic resistance [107,108]. A recent study characterized in detail the consequences of Menin mutations in the interaction with Menin inihbitors [93]. The crystal structure of the Menin mutants T349M, M327I, G331R, and G331D and the N-terminal of MLL1 showed that drug-resistant mutations in Menin occur at a site located in proximity of the MLL1 binding site, but do not affect MLL1 binding to Menin. All of these point mutations in Menin generate a steric collision with Menin inhibitors; the G331D mutant shows a particularly slow dissociation of MLL1 from Menin and thus seems to be particularly difficult to inhibit with small molecule inhibitory drugs [109].

The identification of *MEN1* mutations in AML patients undergoing treatment with Revumenib is important because it may offer opportunities for patients with some *MEN1* mutations to derive benefit from treatment with some new Menin inhibitors.

#### 4.3.2. Bleximenib

JNJ-75276617 (Bleximenib) is a novel potent inhibitor of the protein–protein interaction between Menin and KMT2A; in *KMT2A*-rearranged and *NPM1*-mutant leukemia cells, this compound inhibited the interaction of the Menin–KMT2A complex with chromatin at the level of target genes, resulting in the reduced expression of several target genes such as MEIS1 and FLT3 and exerted potent antiproliferative activity [110]. Bleximenib displayed synergistic antileukemic activity with Gilteritinib, Venetoclax, and Azacitidine [110]. Interestingly, Bleximenib displayed strong antiproliferative activity in leukemic cells bearing mutations (*MEN1^M371^* or *MEN1^T349M^*) observed in patients refractory to Revumenib [110]. In these mutant AMLs, Bleximenib was still able to displace KMT2A and prevent its interaction with Menin despite the presence of *MEN1* mutations that block the activity of other Menin inhibitors.

A first-in-human phase I clinical study evaluated the safety and, preliminary, the efficacy of Bleximenib in 58 R/R acute leukemia patients (56 AML and 2 ALL), showing an acceptable safety profile and preliminary evidence of antileukemic efficacy and biologic activity [111].

A phase I clinical study (NCT 04811560) carried out in 121 R/R acute leukemia (mostly AML, at 108) patients identified the optimal dose of Bleximenib (100 mg) in monotherapy to be used in phase II studies; at this dose, the ORR was 50% and the CR + CRi was 40% [112]. The enrolled patients had either *KMT2A* rearrangements or *NPM1* mutations. Another recent phase I clinical study explored Bleximenib in association with standard chemotherapy in a group of 22 newly diagnosed AML patients (11 *KMT2A*-rearranged and 11 *NPM1*-mutated), showing an acceptable safety profile with no DS or dose-limiting toxicities [113]. The ORR was 93% (83% in *KMT2A*-rearranged AML and 100% in *NPM1*-mutated AML); six patients proceeded to HSCT [113]. A phase Ib (NCT 05453903) clinical study explored the safety and efficacy of Bleximenib in combination with Venetoclax and Azacitidine in R/R AML patients with *KMT2A* rearrangements or *NPM1* mutations [98]. In the safety dataset, 45 patients received the triplet combination treatment, with a median of two prior lines of treatment, including prior Venetoclax treatment in 56% of cases and allo-HSCT in 27% of cases [113]. The safety profile was acceptable and no patient developed DS or tumor lysis syndrome [114]. In the efficacy dataset, the ORR was 86% and the rate of CR + CRi was 48%; for patients with prior Venetoclax exposure, the ORR was 82% and the rate of CR + CRi was 36% [114]. Nine responder patients discontinued treatment and proceeded to allo-HSCT [114] (Table 5).

Hogeling et al. recently reported a large in vitro screening of primary AML cells incubated in the presence of Bleximenib [115]. This study showed that AML cells bearing *NPM1* mutations or *KMT2A* rearrangements are sensitive to the antiproliferative and differentiation-inducing effects of Bleximenib [115]. Concerning *NPM1*-mutant AMLs, those bearing both *NPM1* and *DNMT3A* mutations are those markedly sensitive to the induction of differentiation [104]. In addition to these AML subtypes, AML characterized by a granulo-monocyte progenitor (GMP)-like phenotype also displayed sensitivity to Bleximenib; these cases included leukemias bearing CEBPA mutations [115]. Gene expression studies have shown that AMLs responding to Bleximenib display some epigenetic alterations inducing a striking upregulation of HLA class I and class II expression through a mechanism independent of *MEIS 1* loss [115]. These epigenetic changes resulted in an enhanced sensitivity of leukemic cells to T cell-mediated cytotoxicity in allogeneic and autologous settings [115].

In line with this observation, previous studies have shown that CEBPA is an essential collaborator in HOXA9/MEIS 1-mediated leukemogenesis [116]; CEBPA and KMT2A are co-localized on chromatin and CEBPA-mutated HPCs are hypersensitive to the pharmacologic targeting of the KMT2A complex using Menin inhibitors [117].

#### 4.3.3. DSPP-5336 (Enzomenib)

Enzomenib is a potent, orally bioavailable inhibitor of the Menin–MLL interaction, exhibiting antiproliferative activity in vitro in cell lines, primary leukemic blasts, and patient-derived xenograft mouse models of *KMT2A*-rearranged or *NPM1*-mutant AMLs [118]. Enzomenib directly binds to Menin (Kd 6.0 nM) and inhibits the Menin–KMT2A interaction (IC_50_ 1.4 nM) [118].

A phase I/II clinical study evaluated 81 patients in Arm A (without CYP3A4 inhibitor) and in Arm B (with CYP inhibitor) for the safety and efficacy of a treatment based on Enzamenib monotherapy [119]. In the phase I portion of the study, it was found that the active doses of Enzomenib corresponded to ≥ 140 mg [120]. In total, 35 patients were treated at the active Enzomenib dose: 22 patients had *KMT2A*-rearranged AML (20 AML and 2 ALL) with an ORR of 59% and a CR + CRi of 22.7%; 13 *NPM1*-mutant AML patients had an ORR of 53.8% and a CR + CRi of 23% [120]. The pharmacodynamic changes supported the induction of cell differentiation with a decrease in stemness markers (HOXA9 and MEIS1) and an increase in differentiation markers (CD11b) [120]. DS was observed in 11% of the treated patients (Table 5).

#### 4.3.4. KO-539 (Ziftomenib)

Ziftomenib is an oral Menin inhibitor targeting the Menin–KMT2A protein–protein interaction; Ziftomenib is metabolized into two metabolites with comparable activity to Ziftomenib itself. Preclinical studies have supported the antileukemic activity of Ziftomenib in models of *NPM1*-mutant and *KMT2A*-rearranged AMLs. Particularly, Ziftomenib induced Menin protein degradation through the ubiquitin–proteasome pathway, reduces the MEIS1, FLT3, CDK6, and BCL2 protein levels, in association with the induction of differentiation and the induction of cell apoptosis of leukemic cells harboring *MLL*-rearrangements or *NPM1* mutations [121]. Furthermore, co-treatment with Ziftomenib and BCL-2, CDK6, or BET inhibitors induced synergistic lethality in *MLL*-rearranged or *NPM1*-mutant leukemic cells [121]. These findings were confirmed and extended in a second study showing that Ziftomenib had marked anti-proliferative activity in combination with drugs from various classes, including those targeting chromatin regulation and DNA damage as well as apoptosis and cell cycle block [122]. Particularly pronounced was the synergistic interaction between Ziftomenib and Venetoclax, resulting in pronounced anti-proliferative activity in *MLL*-rearranged and *NPM1*-mutant leukemic cells [122].

The KOMET-001 multicenter, open-label, multicohort phase I/II clinical trial evaluated Ziftomenib in adult R/R AML patients; the study was subdivided into two phases: a phase Ia (dose–response), in which the patients received Ziftomenib (from 50 to 1000 mg) orally once daily in 28-day cycles; and a phase Ib, in which patients with *KMT2A* rearrangements or *NPM1* mutations were randomly assigned to two parallel dose cohorts (200 and 600 mg Ziftomenib) [123]. In phase Ib, no responses were observed in the patients treated at 200 mg of Ziftomenib; at the recommended dose for phase II (600 mg), the rate of CR + CRi was 12.5% (2/16) in the *KMT2A*-rearranged and 35% (7/20) in the *NPM1*-mutant AML patients; the ORR in the *KMT2A*-rearranged AML patients was 17% and 45% in the *NPM1*-mutant AML patients; the median OS was 6.0 and 5.6 months, respectively, in the *KMT2A*-rearranged and *NPM1*-mutant AMLs [123].

The KOMET-007 phase I clinical study evaluated the safety and the efficacy of Ziftomenib combined with standard chemotherapy in 34 newly diagnosed AML patients with *KMT2A* rearrangements or *NPM1* mutations; the patients were treated either with 200 or 400 mg of Ziftomenib [124]. For *NPM1*-mutant AML patients, the CR + CRi rate was 100% at 200 mg and 86% at 400 mg, with MRD negativity among the responders of 100% and 80%, respectively; for the *KMT2A*-rearranged patients, the CR + CRi rates were 90% at 200 mg and 63% at 400 mg, with an MRD negativity rate among the responders of 83% and 100%, respectively [124].

The ongoing KOMET-008 trial is an open-label dose-escalation and expansion study aiming to determine the safety, tolerability, and preliminary efficacy of Ziftomenib in association with standard-of-care regimens for the treatment of either R/R *NPM1*-mutant AML (arm A) or *KMT2A*-rearranged AML (arm B). Arm A is subdivided into three cohorts: cohort A-1 (Ziftomeniob + FLAG-IDA), cohort A-2 (Ziftomenib plus LDAC), and cohort A-3 (Ziftomenib plus Gilteritinib in *NPM1/FLT3* double mutant AML). Arm B is subdivided into two cohorts: cohort B-1 (Ziftomenib plus FLAG-IDA) and cohort B-2 (Ziftomenib plus LDAC) [125].

#### 4.3.5. BMF-219 (Icovamenib)

BMF-219 is the only covalent Menin inhibitor in clinical development under evaluation in multiple hematologic malignancies, solid tumors, and diabetes mellitus. Preclinical studies have shown that BMF-219 shows sustained and marked inhibition of Menin-dependent oncogenic signaling.

The phase I COVALENT-101 phase I dose-escalation and dose-expansion study evaluated BMF-219 in R/R acute leukemia (cohort 1), DLBCL (cohort 2), multiple myeloma (Cohort 3), and CLL (cohort 4) [115]. A recent report analyzed the results observed in 26 R/R AL patients (24 AML and 2 ALL) enrolled in two parallel arms, with or without a CYPP314 inhibitor [126]. BMF-219 was usually well tolerated, with no dose-limiting toxicities; DS was observed in 13% of the cases [115]. Five patients were evaluable for response and two achieved CR (one CR and one CRi) [126].

#### 4.3.6. Menin Inhibitors in AML Patients with NUP98 Rearrangements

Recurrent chromosomal rearrangements involving the Nucleoporin 98 (NUP98) gene, detected in 5–10% of pediatric AML cases and in 2–4% of adult AMLs, are classified as high-risk AMLs [116]. In NUP98 rearrangements, the NUP98 gene is fused with various partners, the most frequent being NSD1 and KDM5A [127]. NUP98 fusions elicit leukemogenesis through interactions with histone-modifying complexes: particularly, NUP98 fusion proteins interact with KMT2A complexes and are co-localized with KMT2A at the level of *HOX A/B* genes [128]. *NUP98*-rearranged AMLs are Menin-dependent, as supported by the observation that the Menin inhibitor VTP50469 induced antiproliferative effects and prolongation of survival in mouse AML models driven by NUP98 fusion proteins [129].

Another study showed that the Menin inhibitor Revumenib inhibited the proliferation and survival of primary NUP08 fusion protein-positive AML cells and inhibited numerous NUP fusion protein target genes, such as MEIS1 and CDK6 [130].

In order to exert their leukemogenetic activity, NUP98 fusion proteins and KMT2A–Menin antagonize the noncanonical polycomb repressive complex 1.1 (PRC1.1) [131].

Interestingly, Carraway et al. reported the case of a patient with NUP98 fusion who relapsed after prior treatments, including allo-HSCT; the patient was treated with BMF-219 and achieved CR after a few cycles of treatment; unfortunately, after 5 months of treatment, the patient relapsed with *NUP98-NSD1*-positive AML [132].

The findings of this case report suggest that Menin inhibitor monotherapy is not sufficient to obtain complete eradication of leukemic clones in *NUP98* fusion-positive AML patients and that the cooperation between Menin inhibition and kinase inhibitors targeting either CDK6 or FLT3 strongly cooperate in *NUP98-*rearranged primary AML cells and in PDX models [133].

In conclusion, the studies carried out using Menin inhibitors have supported their efficacy in monotherapy in *NMP1-*mutant and *KMT2A*-rearranged AMLs. Given the preliminary stage of development of clinical studies with Menin inhibitors, Revumenib, Bleximenib, Enzomenib, and Ziftomenib, at the moment, it is difficult to perform a comparison of their clinical efficacy. Preliminary data have shown the promising efficacy of either Revumenib or Bleximenib in association with an HMA and Venetoclax in R/R *NMP1-*mutant and *KMT2A*-rearranged AMLs; these findings need to be confirmed in a large number of patients and compared to an appropriate control representing a standard of care for these patients.

## 5. Conclusions

In AML, different genetic events determine a block in the differentiation of leukemic cells at various stages of the differentiation/maturation process. Targeted therapies developed in the late twentieth century have introduced a new therapeutic approach based on the targeting of a specific abnormality present in a subset of AML cells. One of the aims of target therapy consists of forcing leukemic cells to differentiate. A prototype of successful differentiation therapy was represented by the treatment of APL using the differentiation-inducing agents ATRA and ATO that have completely revolutionized the therapy of this AML subtype, transforming its outcome from the deadliest to the most curable.

However, ATRA and ATO resulted as not effective in AML outside APL, thus providing evidence that the differentiation block observed in various AML subtypes does not have a uniform underlying mechanism causing differentiation impairment. Thus, the idea that a strategy to induce differentiation in other AML subtypes through the targeting of specific alterations during the leukemic process emerged. This view was strongly supported by recent studies showing that IDH inhibitors, LSD1 inhibitors, and Menin inhibitors induce the differentiation of some AML subtypes. Although these agents have shown a marked capacity to induce leukemic cell differentiation associated with antileukemic effects, therapeutic success was clearly lower than that observed using ATRA + ATO in APL. This lower efficacy of differentiation therapy based on IDH, LSD1, and Menin inhibitors compared to ATRA + ATO in APL is seemingly related to a higher genetic complexity and heterogeneity of *IDH*-mutant, *KMT2A-*rearranged, and *NPM1*-mutant AMLs compared to APL cells, in which all the pathogenic events are driven by PML-RARA fusion protein.

However, in spite of these difficulties, these agents represent an important therapeutic tool for improving the outcomes of *IDH*-mutant, *NPM1-*mutant, and *KMT2A*-rearranged AMLs and possibly also of other AML subtypes. Optimal responses induced by IDH, LSD1, and Menin inhibitors may be significantly improved by strategic partnering with other therapies: ongoing and future clinical studies will clarify the impact of these association therapies, appropriately extended to AML patients at various disease stages. In this context, the introduction of some of these agents into the first-line treatment of elderly non-fit patients using triplet drug combinations is promising.

Future studies will be required to evaluate the impact of these agents on the overall survival in the context of various therapeutic settings involving R/R and first-line patients. Future studies will also assess the potential therapeutic impact of these drugs as maintenance therapy or treatment for the eradication of MRD.

## Figures and Tables

**Table 1:** IDH, LSD1, and Menin inhibitors used in the treatment of AML patients.

Compound	Target	Molecular Structure	Mechanism of Action	Major Effects on Tumor Target	Registration Status
Ivosidenib (AG-120)	IDH1 It inhibits mutant IDH1 at much lower doses than WT IDH1	Small molecule inhibitor (MW 583)	Inhibition of binding pocket of IDH1 dimers	Reduction in 2-HG levels, inhibition of cell proliferation, and induction of cell differentiation	Approved for R/R IDH1-mut AML patients
Olutasidenib (FT-2102)	IDH1 Selective inhibitor of mutant IDH1; no inhibition of WT IDH1	Small molecule inhibitor (MW 355)	Inhibition of binding pocket of IDH1 dimers	Reduction in 2-HG levels, inhibition of cell proliferation, and induction of cell differentiation	Approved for R/R IDH1-mut AML patients
Enasidenib (AG-221)	IDH2 It inhibits mutant IDH2 (R172K, R172S, R140Q) at much lower doses than WT IDH2	Small molecule inhibitor (MW 569)	Inhibition of binding pocket of IDH2 dimers	Reduction in 2-HG levels, inhibition of cell proliferation, and induction of cell differentiation	Approved for R/R IDH2-mut AML patients
Iadademstat (ORY-1001)	LSD1 Highly selective covalent LSD1 inhibitor	Small molecule inhibitor (MW 230)	Inhibition of both demethylating activity of LSD1 and scaffolding function	Reduced proliferation of blast leukemic cells and induction of their differentiation	Orphan Drug-Designated
Ravumenib	Menin	Small molecule inhibitor (MW 841)	Inhibition of Menin-MLL binding	It impairs proliferation and induces cell differentiation in MLL-r and NPM1-mut AML	Approved for R/R AML
Bleximenib (JNJ-75276617)	Menin	Small molecule inhibitor (MW 600)	Inhibition of Menin-MLL binding	It impairs proliferation and induces cell differentiation in MLL-r and NPM1-mut AML	Under investigation
Enzomenib (DSP-5336)	Menin	Small molecule inhibitor (MW 590)	Inhibition of Menin-MLL binding	It impairs proliferation and induces cell differentiation in MLL-r and NPM1-mut AML	Under investigation
Ziftomenib	Menin	Small molecule inhibitor (MW 718)	Inhibition of Menin-MLL binding	It impairs proliferation and induces cell differentiation in MLL-r and NPM1-mut AML	Under investigation

**Table 2:** Major clinical trials involving the treatment of AML patients with Ivosidenib or Olutasidenib alone or in combination with Azacitidine. Abbreviations: ND (newly diagnosed), R/R (relapsed/refractory), CR (complete remission), CRi (complete remission with incomplete hematological response), ORR (overall response rate), BID (two times a day), QD (once a day), EFS (event-free survival), OS (overall survival), MDR (median duration of response).

NCT Identifier Phase	Patient Number and Disease Status	Therapeutic Regimen	Efficacy	Toxicity
NCT02074839 Phase I	179 R/R *IDH1*-mut AML	Ivosidenib 500 mg QD (single arm)	ORR 41.6% CR 21.6% CR + CRi 30.4% MDR 6.5 mo Among responders: 7% IDH1-mut negative	QT interval prolongation 7.8% Differentiation syndrome 3.9% Anemia 2.2% Thrombocytopenia 3.4 Leukocytosis 1.7%
NCT02074839 Phase I	34 ND *IDH1*-mut AML not eligible for standard therapy	Ivosidenib 500 mg QD (single arm)	CR 30.3% CR + CRi 42.4% Median OS 12.6 mo 77.8% of responding patients in remission at 1 year	Differentiation syndrome 9% Anemia 12% Thrombocytopenia 15%
NCT03173248 Phase III	146 ND *IDH1*-mut AML	Azacitine (75 mg/m^2^) Ivosidenib (500 mg/QD) vs. Azacitidine + placebo	ORR 62% vs. 19% CR + CRi 58% vs. 19% EFS at 12 mo 38% vs. 11% mEFS 22.9 vs. 4.1 mo mOS 24 vs. 7.9 mo	Differentiation syndrome 4% vs. 4% Febrile neutropenia 20% vs. 34% Thrombocytopenia 20% vs. 15% Infection 21% vs. 30%
NCT02719574 Phase I/II	126 (expansion cohort) R/R *IDH1*-mut AML	Olutasideb 150 mg BID (single arm)	0RR 48% CR + CRi 35% mOS 11.6 mo In responding patients mOS Not reached MDR 11.6 mo MDR in responding patients 25.9 mo	Differentiation syndrome 9% Febrile neutropenia 20% Thrombocytopenia 16% Anemia 16%
NCT02719524 Phase I/II	67 R/R *IDH1*-mut AML	Olutasideb 150 mg BID + Azacitidine (75 mg/m^2^) (sigle arm)	ORR 51% CR + CRi 31% mOS 12.5 mo mOS in patients achieving CR + CRi 36 mo	Anemia 25% Thrombocytopenia 37% Febrile neutropenia 19% Leukocytosis 6%

**Table 3:** Major clinical trials involving the use of Enasidenib in AML patients, either alone or in combination with Azacitidine or Venetoclax.

NCT Identifier Phase	Patient Number and Disease Status	Therapeutic Regimen	Efficacy	Toxicity
NCT01915498 Phase I/II	153 (expansion phase) R/R IDH2-mut AML	Enasidenib 100 mg QD (single arm)	ORR 38.5% CR + CRi 26.6% MDR 5.6 mo MDR in CR 8.8 mo mOS 9.3 mo mOS in CR 19.3 mo	Differentiation syndrome 7% Hyperbilirubinemia 8%
NCT02577406 Phase I	319 R/R IDH2-mut AML	Enasidenib 100 mg QD vs. Conventional therapy	ORR 40.5% vs. 9.9% CR 23.4% vs. 3.7% CR + CRi 29.7% vs. 6.2% OS at 12 mo 38% vs. 26% mEFS 4.9 mo vs. 2.6 mo	Differentiation syndrome 5% vs. 0.0% Hyperbilirubinemia 10.8% vs. 0.0%
NCT03013998 Phase Ib/II	60 ND IDH2-mut AML not suitable for standard therapy	Total of 60 ND IDH2-mut AML patients treated with 5 cycles of Enasidenib: patients with CR + CRi continued Enasidenib, patients not responding were treated with Ena + AZA Enasidenib 100 mg QD (phase II) Enasidenib + Azacitidine (75 mg/m^2^) (phase Ib)	Phase II vs. phase Ib CR + CRi 48% vs. 40% MDR 11.1 mo vs. 14.6 mOS 17.1 mo vs. 12.5 mo at 24 months, mOS 41% vs. 47%	Differentiation syndrome 20% vs. 11.8% Thrombocytopenia 5% vs. 29% Anemia 5% vs. 17% Leukopenia 3% vs. 33%
NCT04092179 Phase II	27 R/R IDH2-mut AML or MDS	Enasidenib 100 mg QD BID (single arm) Venetoclax 400 mg QD	0RR 70% CR 57% Responses were higher in IDH2R140 than in IDH2R172-mutant patients	Febrile neutropenia 41% Thrombocytopenia 26% Hyperbilirubinemia 48% Leukocytosis 8%

**Table 4:** Clinical trials involving the LSD1 inhibitor Iadademstat.

Compound	NCT Identifier	Patient Number	Patient Typology	Toxicity	Efficacy
Iadademstat (ORY-1001)	EDRA CT 2013-002447-29	27 dose-escalation (5–220 μg/m^2^/day) 14 dose-expansion (140 ug/m^2^/day)	R/R AML	Myelosuppression and non- hematological adverse events (infections, asthenia, mucositis diarrhea) Differentiation syndrome two patients. One grade 3 CS and One patient was fatal	2CRi Blast reduction and cell differentiation in 2/4 MLL-rearranged and 2/4 erythroleukemia
Iadademstat (ORY-1001) Azacitidine	Phase II	60 or 90 mg/m^2^/day	Unfit AML patients	Myelosuppression with anemia, Thrombocytopenia, and granulocytpenia One grade 3 DS and one fatal grade 5 intrachranial hemorrhagia	ORR of 81% in responding patients and 64% CR and 36% PR Responding AML subtypes: Flt3-mutated (3/3), TP53-mutated (75%), FABM 4/M5 86%
Iadademstat (ORY-1001) Gilteritinib	PhaseI/II	Dose-escalation from 75 to 150 μg/m^2^/day 13 patients at 75 or 100 μg/m^2^/day	FLT3-mutant R/R AML	Not reported	At 100 μg/m^2^/day, 5/6 patients cleared BML blasts; at 75 μg, 2/7 had no response, 1/7 CR, and 1/7 CRi; 1 patient cleared BM blast; and 2 patients not yet assessed

**Table 5:** *KMT2A*-rearranged and *NPM1*-mutant AML patients treated with Menin inhibitors in monotherapy.

Compound	Revumenib	Bleximenib	Enzomenib	Ziftomenib
Trial	AUGMENT-101 Phase I/II	CAMELOT-1 Phase I/II	DSP-5336-101 Phase I/II	KOMET-001 Phase I/II
Number of patients	161	21	40	58
ORR	KMT2Ar 64% NPM1m 47%	KMT2Ar 30% NPM1m 50%	KMT2Ar 59% NPM1m 54%	KMT2Ar 17% NPM1m 42%
CR + CRi	KMT2Ar 23% NPM1m 23%	KMT2Ar 33% NPM1m 33%	KMT2Ar 30% NPM1m 47%	KMT2Ar 17% NPM1m 35%
MRD negativity (in CR + CRi)	KMT2Ar 58% NPM1m 64%	NR	NR	KMT2Ar 100% NPM1m 63%
HSCT among responders	KMT2Ar 36% NPM1m 17%	NR	NR	KMT2Ar 33% NPM1m 33%
Differentiation Syndrome (%)	22%	19%	11%	11%
